# Local and systemic transcriptome and spliceome reprogramming induced by the root-knot nematode *Meloidogyne incognita* in tomato

**DOI:** 10.1093/hr/uhae206

**Published:** 2024-07-26

**Authors:** Selin Ozdemir, Sarbottam Piya, Valeria S Lopes-Caitar, Nicole Coffey, J Hollis Rice, Tarek Hewezi

## Abstract

Root-knot nematodes (*Meloidogyne* spp.) are widely spread root parasites that infect thousands of vascular plant species. These highly polyphagous nematodes engage in sophisticated interactions with host plants that results in the formation of knot-like structures known as galls whose ontogeny remains largely unknown. Here, we determined transcriptome changes and alternative splicing variants induced by *Meloidogyne incognita* in galls and neighboring root cells at two distinct infective stages. *M. incognita* induced substantial transcriptome changes in tomato roots both locally in galls and systemically in neighboring cells. A considerable parallel regulation of gene expression in galls and neighboring cells were detected, indicative of effective intercellular communications exemplified by suppression of basal defense responses particularly during the early stage of infection. The transcriptome analysis also revealed that *M. incognita* exerts a tight control over the cell cycle process as a whole that results in an increase of ploidy levels in the feeding sites and accelerated mitotic activity of the gall cells. Alternative splicing analysis indicated that *M. incognita* significantly modulates pre-mRNA splicing as a total of 9064 differentially spliced events from 2898 genes were identified where intron retention and exon skipping events were largely suppressed. Furthermore, a number of differentially spliced events were functionally validated using transgenic hairy root system and found to impact gall formation and nematode egg mass production. Together, our data provide unprecedented insights into the transcriptome and spliceome reprogramming induced by *M. incognita* in tomato with respect to gall ontogeny and nematode parasitism.

## Introduction

Root-knot nematodes (*Meloidogyne* spp.) are obligatory root parasites that infect almost all vascular plant species including many economically important crops, vegetables, and ornamental plants, causing significant economic losses [1]. With over 100 species of *Meloidogyne* species described so far [2], *Meloidogyne incognita* is the most globally widespread and damaging species. These highly polyphagous nematodes engage in sophisticated interactions with host plants that results in the formation of a permanent feeding site, known as giant-cells, to support nematode development and maturation. The infective second-stage juveniles (J2) penetrate plant roots at the root tip region, migrate intercellularly, and select few vascular cylinder cells as potential feeding cells. If these cells are compatible, the infective J2s become sedentary and guide these cells to enlarge in size via recurrent mitosis without cytokinesis to form multinucleated and metabolically hyper-active giant-cells [3, 4]. Giant-cell formation stimulates neighboring cells to divide asymmetrically in a disordered way, resulting in the formation of knot-like swellings termed galls at the site of infection. The infective J2s feed from the giant-cells and molts to third-stage juveniles (J3), fourth-stage juveniles (J4), and finally adult females laying hundreds of eggs inside the egg [1, 3].

The identity of nematode-induced galls and giant-cells is believed to be established through specific transcriptional reprogramming, which is mediated via a broad set of transcription factors in addition to other transcriptional and post-transcriptional regulators. *M. incognita*–induced transcriptome programming in host plants has been documented in several studies [5–14], providing interesting information about the importance of phytohormone signaling, suppression of defense responses, cytoskeletal organization, solute transport, cell wall modifications, regulation of metabolic pathways, for successful nematode parasitism of host plants. However, most of these transcriptome studies were conducted using whole root tissues, and hence lack the fine resolution required for deepening our understanding of cellular responses to *M. incognita* infection and the molecular mechanisms underlying gall formation and development.

Post-transcriptional regulation of mRNA by alternative splicing (AS) has recently emerged as an important regulatory mechanism determining gene function and specificity in various developmental and stress contexts [15–19]. Exon skipping and intron retention, the two main splicing events, impact transcriptome diversity and function. For example, the generation of multiple mature mRNA transcripts from a single pre-mRNA through combinatorial exclusion or inclusion of exons has the potential to produce protein isoforms with varied structural and functional specificities. Intron-retention events frequently produce non-functional transcripts that could play a role in fine-tuning the transcript level of certain disease resistance genes to reach appropriate activity and maximize plant fitness [19–21]. In some cases, intron-retained transcripts can be translated, producing truncated proteins that may have dominant-negative functions [18, 20, 22].

Despite recent experimental data indicate that AS plays fundamental role in various pathosystems [17, 19], information about the frequency and importance of AS events during plant-nematode interactions has been reported in only a limited number of studies [23–26]. For example, soybean cyst nematode (SCN, *Heterodera glycines*) has been shown to induce 1979 splicing events in 1576 unique genes in soybean roots upon infection with exon skipping being the most common differentially spliced events [26]. Importantly, effectors from cyst and root-knot nematodes have been shown to interacts with host spliceosomal proteins in order to reprogram plant mRNA splicing machinery and facilitate infection. The 30D08 effector from the beet cyst nematode *Heterodera schachtii* and the MiEFF18 effector from *M. incognita* were reported to physically associate with the auxiliary spliceosomal SMU2 and SmD1 from Arabidopsis, respectively, thereby modulating the splicing patterns of plant genes to facilitate nematode infection [23, 24]. Despite these compelling evidences, the magnitude, patterns, and functions of differentially spliced events occurring during the formation and development of *M. incognita*–induced galls remain elusive.

In this study, we determined transcriptome changes and alternative splicing variants, induced by *M. incognita* in galls and neighboring root cells at two distinct infection stages. Our data indicate that *M. incognita* induces robust gene expression changes in tomato roots both locally in galls and systemically in neighboring cells. This transcriptome reprograming occurred in an infection stage-dependent manner and involved parallel regulation in galls and neighboring cells. Our analysis also revealed that *M. incognita* greatly modulates pre-mRNA splicing particularly during the early stage of infection as a total of 9064 uniquely differentially spliced events from 2898 genes were identified. The functional importance of certain splicing variants was examined using transgenic hairy root system and found to alter nematode parasitism by impacting gall formation and nematode egg mass production. Overall, our results shed light on how *M. incognita* alters host gene expression and pre-mRNA splicing to regulate various biological processes and molecular functions required for gall organogenesis and nematode parasitism.

## Results

### *M. incognita* induces substantial transcriptome changes in tomato roots both locally in galls and systemically in neighboring cells

To determine local and systemic transcriptome changes induced by *M. incognita* in tomato roots, we inoculated 10-day-old tomato seedings with second-stages juveniles. At 4- and 11-day post inoculation (dpi), galls, neighboring roots tissues, and the corresponding non-infected whole roots we collected in three biological samples for RNA extraction and RNA-seq library preparation (Supplementary Fig. S1). The 4- and 11-day time points were selected to represent young and fully developed galls. Using a false discovery rate less than 5%, we identified 3895 and 6944 differentially expressed genes (DEGs) in galls and adjacent root tissues, respectively, in comparison with non-infected roots at 4 dpi (Supplementary Data Table S1 and S2). Similarly, at 11 dpi 3582 and 1063 DEGs were identified in galls and adjacent root tissues, respectively, in comparison with the corresponding non-infected roots (Supplementary Data Table S3 and S4). These data indicate that *M. incognita* induces robust gene expression changes in tomato roots both locally in galls and systemically in neighboring cells.

### *M. incognita* induces transcriptome reprograming in an infection stage-dependent manner

**Figure 1:**
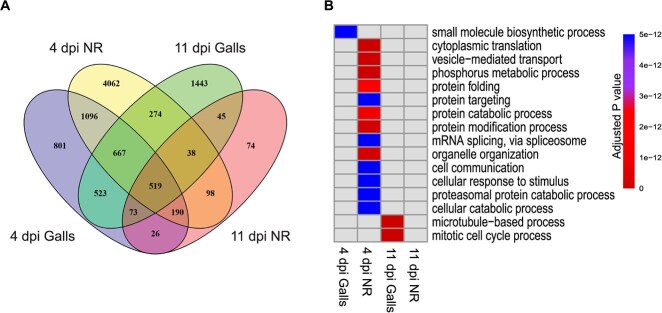
*Meloidogyne incognita* induces transcriptome reprograming in an infection stage-dependent manner. A: Venn diagram showing overlapping and uniquely differentially expressed genes (DEGs) between galls and neighboring root cells (NR) at 4 and 11 dpi. B: Heat map representation of GO terms significantly enriched among DEGs unique to galls and neighboring root cells at 4 and 11 dpi. GO term enrichment analysis was performed using PANTHER version 18.0 with Fisher's exact test and Bonferroni multi-test adjustment with a cutoff P-value of 0.01 for significance.

We examined whether *M. incognita* induces distinct transcriptome reprograming at the 4 and 11 dpi. Venn diagrams revealed that 801 and 1443 genes were uniquely differentially expressed in galls at the 4 and 11 dpi time points, respectively (Fig. 1A). Likewise, 4062 and 74 genes were exclusively differentially expressed in the neighboring root tissues at the 4 and 11 dpi time points, respectively (Fig. 1A). This finding implies that *M. incognita* induces transcriptome reprograming in galls and neighboring cells in an infection stage-dependent fashion. The 4-dpi gall specific DEGs (801) were enriched in genes involved in small molecule biosynthetic process, whereas the 11-dpi gall-specific DEGs (1443) were enriched in genes implicated in mitotic cell cycle process and microtubule-based process. The DEGs unique to the neighboring cells at 4 dpi (4062) were enriched in genes implicated in cytoplasmic translation, cellular catabolic process, vesicle-mediated transport, phosphorus metabolic process, protein folding, targeting, and modification, mRNA splicing, organelle organization, cell communication, and cellular response to stimulus (Fig. 1B). The DEGs unique to the neighboring cells at 11 dpi (74) did not show enrichment in a particular biological process (Fig. 1B).

### *M. incognita* induces parallel transcriptome reprograming in galls and neighboring cells

**Figure 2:**
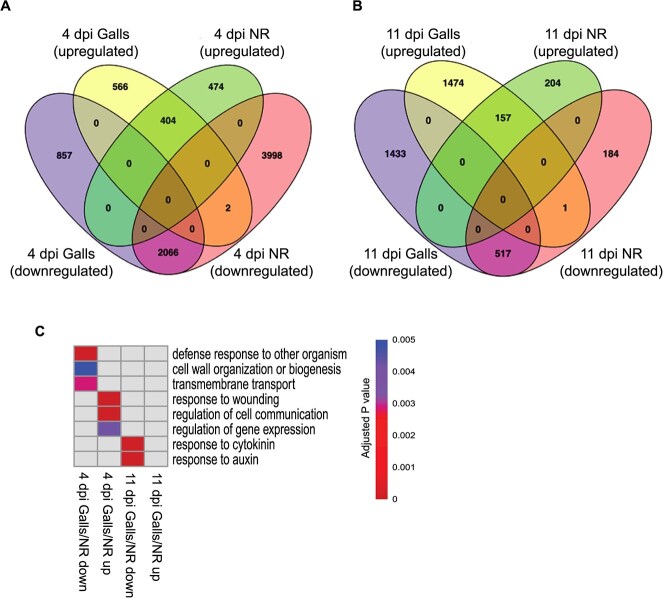
*Meloidogyne incognita* induces parallel transcriptome reprograming in galls and neighboring cells. A and B: Venn diagrams of differentially upregulated and downregulated genes identified in galls and neighboring cells at 4 (A) and 11 dpi (B). C: Heat map representation of GO terms significantly enriched among similarly regulated genes in galls and adjacent roots tissues at 4 and 11 dpi. GO term enrichment analysis was performed using PANTHER version 18.0 with Fisher's exact test and Bonferroni multi-test adjustment with a cutoff P-value of 0.01 for significance.

Cross comparison of the identified DEGs revealed that 2470 (2066 + 404) and 674 (517 + 157) genes were similarly up- or down-regulated in galls and adjacent root tissues at 4 and 11 dpi, respectively (Fig. 2 A and B). Only three genes at these two time points showed opposite regulation in galls and neighboring root tissues (Fig. 2A and B). The finding that 63.5% of the DEGs identified in galls at 4 dpi were similarly regulated in neighboring root tissues implies that this parallel regulation is not random but is biologically meaningful. Interestingly, gene ontology (GO) analysis of the 2066 downregulated genes both in galls and in neighboring cells at 4 dpi were enriched in genes involved in defense response to other organisms, as well as those implicated in cell wall organization or biogenesis and transmembrane transport (Fig. 2C). Protein class analysis revealed that water and metal ion transmembrane transporters and peroxidases were highly overrepresented among these downregulated genes. The similarly upregulated genes (404) in galls and in neighboring root tissues at 4 dpi were enriched in genes involved in wounding response, regulation of cell communication and regulation of gene expression (Fig. 2C). Remarkably, protein class analysis of these 404 upregulated genes revealed enrichment for transcription factors and metabolite interconversion enzymes, particularly those with hydrolase, oxidoreductase, phospholipase, and oxygenase functions. The 674 genes that were similarly regulated in galls and adjacent roots tissues at 11 dpi included 517 downregulated genes and 157 upregulated genes. Response to cytokinin and response to auxin were the significantly enriched GO terms among the 517 downregulated genes (Fig. 2C). The 157 similarly upregulated genes in galls and neighboring regions were involved in a wide range of biological processes but no such enrichment for GO terms was detected. Together, these gene expression data point to inhibition of auxin and cytokinin signaling and defense responses locally in the nematode-inducted galls and systemically in the neighboring cells.

### Transcription factors regulatory networks controlling gene expression in galls and neighboring regions

**Figure 3:**
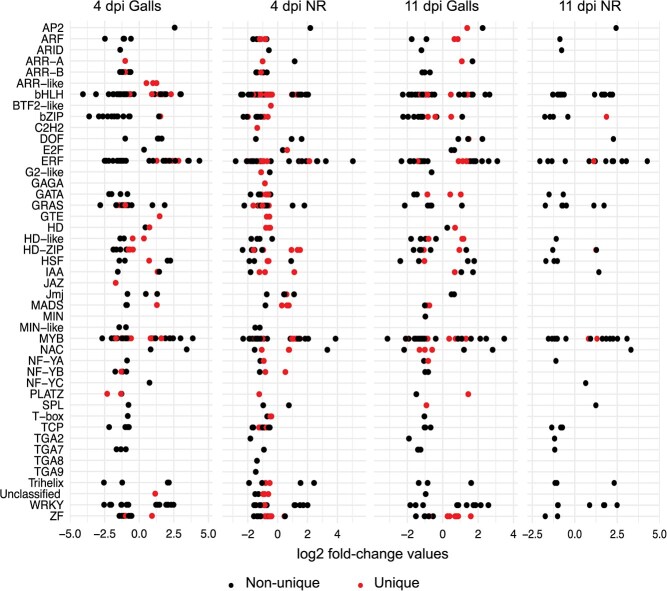
Classification of differentially expressed transcription factors identified in galls and adjacent roots cells at 4 and 11 dpi. Shown are fold change values of differentially expressed transcription factors belonging to various families/subfamilies. Each transcription factor is represented by a dot. Black dots denote transcription factors that were similarly regulated in galls and adjacent roots cells at the 4 or 11 dpi time points. Red dots denote transcription factors that were specifically regulated in galls or adjacent roots cells at 4 or 11 dpi.

The identification of substantial numbers of commonly and uniquely regulated genes between galls and adjacent roots tissues at both time points prompted us to identify transcription factors with potential roles in this regulation. A total of 261 and 319 transcription factor-encoding genes were identified as differentially expressed in galls and adjacent root tissues, respectively at 4 dpi (Supplementary Data Table S5). Similarly, at 11 dpi, 212 and 90 transcription factors were determined as differentially expressed in galls and adjacent root tissues, respectively (Supplementary Data Table S5). After eliminating duplicates, a total of 487 unique transcription factors were identified as differentially expressed in galls and/or adjacent root cells at both time points. As shown in Fig. 3, 236 of these transcription factors belongs to the bHLH, bZIP, ERF, MYB, and WRKY families, implying key functions of these family members in establishing the regulatory networks controlling gene expression in galls and neighboring regions.

Cross-comparison of the transcription factor gene lists revealed that 162 and 62 transcription factors (highlighted in back dots in Fig. 3) were similarly regulated in galls and adjacent root tissues at the 4 and 11 dpi time points, respectively. These factors belong to various families, including AP2, bHLH, bZIP, GATA, ERF, TCP, TGA, and Trihelix, for example, suggesting roles of these factors in coordinating gene expression in galls and adjacent root cells. Cross comparison also revealed that 256 and 178 transcription factors (highlighted in red dots in Fig. 3) were uniquely regulated in galls or neighboring root tissues at 4 and 11 dpi, respectively. Notably, all members of the E2F, GTE, and HD transcription factor families exhibited unique expression patterns in galls or neighboring root tissues (Fig. 3), suggesting key regulatory functions of these factors in mediating gene expression specificity. Examining the expression patterns of the 487 identified transcription factors revealed that two-thirds of these transcription factors were downregulated (Fig. 3 and Supplementary Data Table S5). Remarkably, all members belonging to the TCP, TGA, HD-like, and NF-YA families were downregulated (Fig. 3). Similarly, the majority of the bZIP, GATA, and GRAS family members were also downregulated (Fig. 3). Together, these data suggest that downregulation of key regulatory factors is critical for gall formation and development.

### Mitotic cell cycle processes are highly activated in fully developed galls

**Figure 4:**
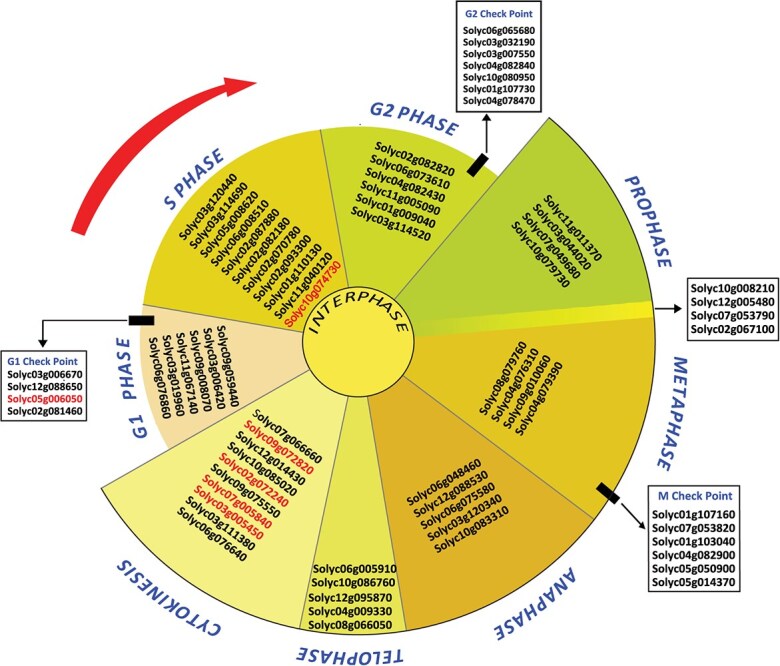
Schematic representation showing distribution of differentially expressed cell cycle-related genes identified in the 11-dpi galls across various cell cycle phases. Upregulated genes are indicated in black, whereas downregulated genes are indicated in red. Gene expression values and annotations are provided in Supplementary Data Table S6.

The enrichment of mitotic cell cycle-related genes among the 11-dpi gall-specific DEGs is remarkable considering the key role of cell cycle in gall ontogeny and nematode parasitism. Therefore, we mapped all differentially expressed cell cycle-related genes, identified in the 11-dpi galls in comparison with the corresponding non-infected roots, across various cell cycle phases. As shown in fig. 4, these genes were distributed across all cell cycle phases including interface phase (G1, S, and G2) and mitotic (M) phase (mitosis and cytokinesis).

The list included several core cell-cycle genes, including the D-type cyclins CYCD1;1 (*Solyc05g006050*) and CYCD6;1 (*Solyc03g006670*), which are involved in G1 phase progression; the DNA replication licensing factors MCM2 (*Solyc11g040120*) and MCM6 (*Solyc02g082180*), DNA REPLICATION FACTOR CDT1 (*Solyc06g008510*), and DNA REPLICATION COMPLEX GINS PROTEIN PSF3 (*Solyc05g008620*) in addition to several G2 mitotic-specific cyclins (*Solyc06g065680*, *Solyc02g082820*, *Solyc06g073610*, *Solyc04g082430*, and Solyc11g005090; Fig. 4 and Supplementary Data Table S6). The core genes of the M phase included KINESIN-like proteins (*Solyc09g010060*, *Solyc06g075580*, and *Solyc10g083310*), CONDENSIN complex subunits (*Solyc07g049680*, *Solyc11g011370*, *Solyc03g044020*, and *Slyc10g079730*), TPX2 like proteins (*Solyc10g008210, Solyc08g079760*, *Solyc12g005480*, *Solyc07g053790*, and *Solyc02g067100*), as well as several tubulins and microtubule binding motor proteins (Fig. 4, and Supplementary Data Table S6). Interestingly, genes involved in cell cycle checkpoints were also identified among the 11-dpi gall-specific DEGs, including, for example, a cyclin-dependent kinase (*Solyc04g082840*), *B-type cyclins* (*Solyc10g080950*, *Solyc01g00904*0, and *Solyc04g078470*), *AURORA kinases* (*Solyc12g095870*, *Solyc04g009330*, and *Solyc08g066050*), a *TRESLIN* (*Solyc03g007550*), a *cell division control protein* (*Solyc09g059440*), and the *mitotic checkpoint proteins BUBR1* (*Solyc04g082900*), *BUB1* (*Solyc07g053820*), and *BUB3* (*Solyc01g107160*) (Fig. 4 and Supplementary Data Table S6).

### Local and systemic suppression of defense response in galls and neighboring root cells

**Figure 5:**
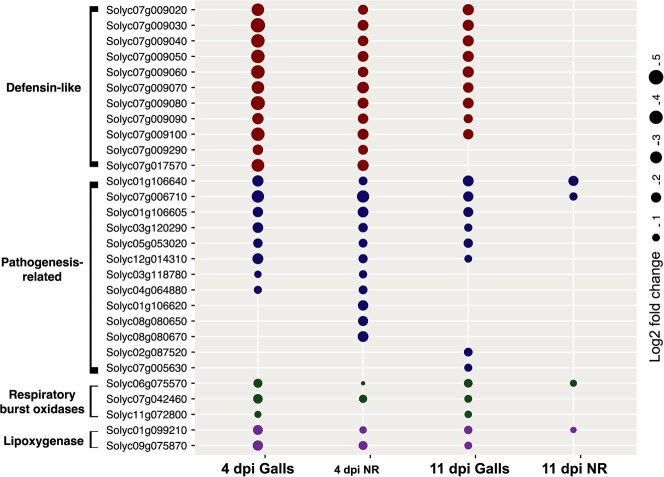
Local and systemic suppression of defense response in galls and neighboring root cells. Fold change plot of downregulated defense-related genes in galls and neighboring root cells at 4 and 11 dpi. Bubble size represents the level of downregulation for each gene.

GO term analysis implied local and systemic suppression of defense responses in galls and neighboring root cells during the early stage of infection. To provide more details for this finding, we compared the levels of downregulation of a set of genes known to be involved in basal defense response between galls and neighboring root cells at both time points. The expression of 11 defensin-like genes were simultaneously downregulated in the 4-dpi samples. However, the level of downregulation in neighboring root cells tended to be at a lower magnitude (Fig. 5).

Additionally, genes encoding pathogenesis-related (PR) proteins, respiratory burst oxidases, and lipoxygenase exhibited the same patterns of downregulation (Fig. 5), supporting the notion of systemic suppression of defense responses upon nematode infection. Nevertheless, at the 11-dpi time point, the majority of these genes showed similar downregulation in galls but not in the neighboring root cells, suggesting a transition of defense suppression from local/systemic state at the early stage of infection into local state at later stage on infection.

It may be important to mention that a number of defense-related genes, including PR genes, defensin-like genes, receptor like kinases, and WRKY transcription factors with known roles in deferens responses were upregulated in galls or neighboring root cells particularly at the 4-dpi (Supplementary Data Tables S1 and S2). The upregulation of these defense-related genes may reflect that tomato plants ineffectively respond to nematode infection or reflect a mechanism to mitigate damage.

### *M. incognita* profoundly modulates pre-mRNA splicing during infection

**Figure 6:**
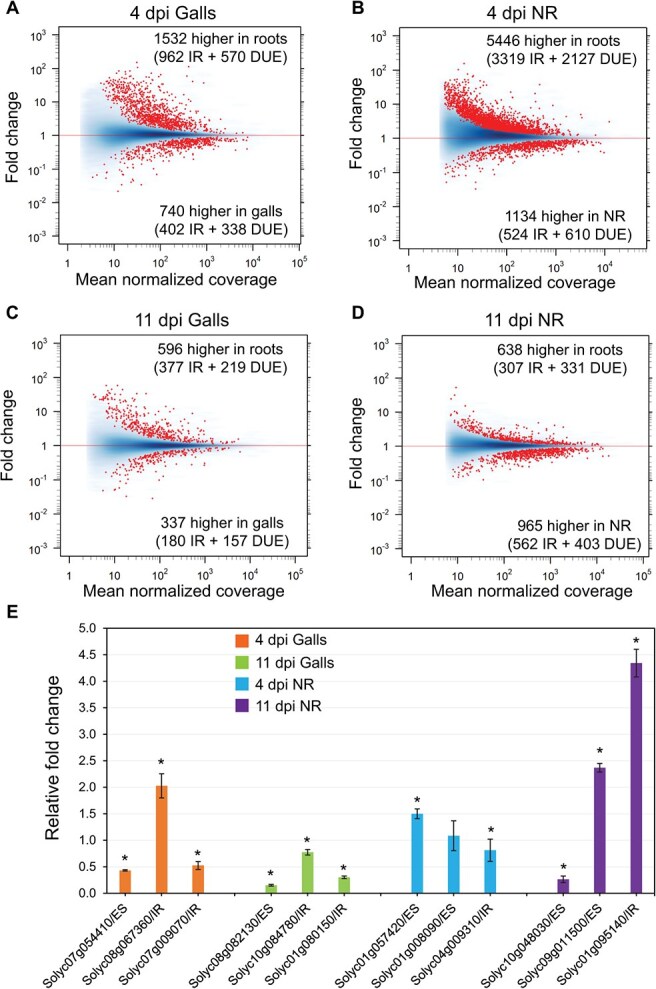
Modulation of pre-mRNA splicing during *Meloidogyne incognita* infection. A-D: MA plots comparing fold change values against the mean of the normalized counts of differentially spliced events determined in galls (A and C) and neighboring region (NR) (B and D) at 4 and 11 dpi. Red dots represent significantly differentially spliced events. DUE: differentially used exons; IR: intron retention. E: RT-qPCR quantification of the expression levels of differentially spliced events in galls and NR at 4 and 11 dpi. Shown are the fold change values of 6 exon skipping (ES) and 6 IR events in galls or NR at 4 and 11 dpi relative to the corresponding non-infected control root tissues. Data are the mean ± SE of three biological replicates.

We next analyzed our RNA-seq data to identify differentially spliced events using the JunctionSeq package [27]. Using a FDR cut-off of 0.05, we identified 2272 and 6580 differentially spliced events in galls and neighboring root tissues, respectively, as compared with non-infected roots at 4 dpi (Supplementary Data Tables S7 and S8, and Fig. 6A and B). Similarly, 933, and 1603 differentially spliced events were identified in galls and adjacent root tissues, respectively, compared with non-infected roots at 11 dpi (Supplementary Data Tables S9 and S10, and Fig. 6C and D). A total of 9064 unique differentially spliced events from 2898 genes were identified after eliminating duplicates. These results indicate that *M. incognita* profoundly modulates pre-mRNA splicing particularly during the early stage of infection.

### *M. incognita* induces suppression of intron retention events and exon usage both in galls and neighboring cells

The differentially spliced events were further grouped into two main categories: intron retention (IR) and differentially used exons (DUEs). Of the 2272 differentially spliced events identified in the 4-dpi galls, 740 events (402 IR and 338 DUE) were significantly more abundant in galls, and 1532 events (962 IR and 570 DUE) were significantly more abundant in the corresponding non-infected root tissues (Fig. 6A). Of the 6580 differentially spliced events identified in the neighboring regions at 4 dpi, 1134 events (524 IR and 610 DUE) were significantly more abundant in neighboring regions, and 5446 events (3319 IR and 2127 DUE) were significantly more abundant in the corresponding non-infected root tissues (Fig. 6B). These data imply that suppression of IR events and increasing exon skipping occur in galls and to a much higher extent in the neighboring cells.

This trend was also observed in galls and neighboring cells at 11 dpi despite the fact that the numbers of differentially spliced events were substantially reduced. The 933 differentially spliced events identified in galls at 11 dpi included 337 events (180 IR and 157 DUE), which accumulated more significantly in galls, and 596 events (377 IR and 219 DUE), which accumulated more significantly in the corresponding non-infected root tissues (Fig. 6C). The 1603 differentially spliced events identified in the neighboring regions at 11 dpi included 965 events (562 IR and 403 DUE), which accumulated more significantly in neighboring regions, and 638 events (307 IR and 331 DUE), which accumulated more significantly in the corresponding non-infected root tissues (Fig. 6D).

To validate IR and DUE data, we used reverse-transcription quantitative PCR (RT-qPCR) to measure the abundance of 6 IR events (*Solyc08g067360*, *Solyc07g009070*, *Solyc10g084780*, *Solyc01g080150*, *Solyc04g009310*, and *Solyc01g095140*) and 6 exon skipping events (*Solyc07g054410*, *Solyc08g082130*, *Solyc01g057420*, *Solyc01g008090*, *Solyc10g048030*, and *Solyc09g011500*) in galls or neighboring cells at 4 or 11 dpi relative to non-infected root tissues. With the exception of the Solyc01g008090 exon skipping event, which did not show significant change in abundance, the expression data of the remaining 11 events confirmed the splicing patterns obtained with JunctionSeq (Fig. 6E).

### Genes involved in oxidation reduction are subjected to alternative splicing in gall and neighboring cells

We next performed GO term enrichment analysis to identify biological processes and molecular functions that are overrepresented among the differentially spliced genes (DSGs) showing increased or decreased IR/exon usage events in galls and neighboring cells at 4 and 11 dpi. At 4 dpi, genes showing increased IR and/or exon usage in galls were enriched in functions related to catalytic activity and wounding response, whereas those showing reduced IR and/or exon usage were enriched in genes related to oxidation–reduction process and transmembrane transporter activity. Similarly, genes showing increased IR and/or exon usage in the neighboring cells at 4 dpi were enriched in functions related to catalytic activity, hydrolase activity, and wounding response. However, genes with reduced abundance of IR and/or exon usage events in the neighboring region at 4 dpi were enriched in biological processes and molecular functions related to cellular lipid metabolic processes, protein and small molecule metabolic processes, macromolecule biosynthetic process, transport, oxidoreductase activity, and RNA binding. At 11 dpi, DSGs with increased IR and/or exon usage in galls showed an enrichment for oxidoreductase activity, while those exhibiting reduced IR and/or exon usage were enriched in oxidoreductase activity and transmembrane transporter activity. DSGs with increased IR and/or exon usage events in the neighboring region at 11 dpi were enriched in functions related to wounding responses and oxidoreductase activity, whereas those genes showing reduced IR and/or exon usage events were enriched in genes related to catalytic activity and primary metabolic process (Supplementary Fig. S2). These data clearly show that a significant number of genes involved in oxidation–reduction are subjected to alternative splicing both in gall and neighboring cells at early and late stages of nematode infection.

### Differentially used exons impact protein domain arrangement

**Figure 7:**
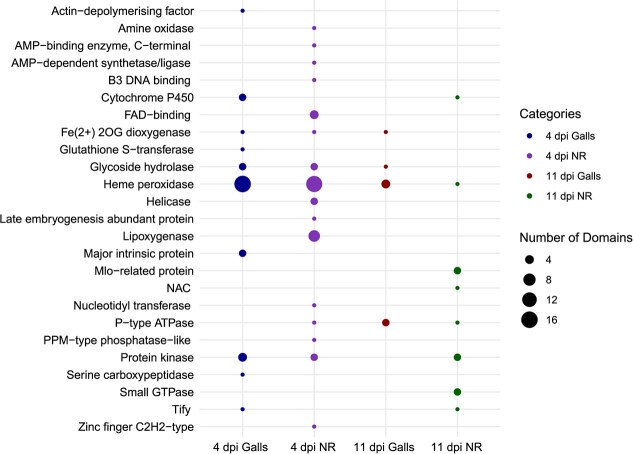
Impact of differentially used exons on protein domain organization. The bubble plot illustrates the most common protein domains identified in the differentially used exons in galls and neighboring root cells (NR) at 4 and 11 dpi. The size of each bubble represents the number of functional protein domains allocated to each group.

Differential usage of exons in specific splicing variants have the potential to impact protein domain organization, and hence protein function [18]. Therefore, we examined if DUEs identified in galls and neighboring cells would affect protein domain structure by scanning the amino acid sequences of significantly DUEs against functionally annotated protein domains in the Pfam database. Numerous functional domains, including cytochrome P450, glycoside hydrolase, peroxidase, peptidase, helicase, S-adenosyl-l-methionine–dependent methyltransferase, and small GTPase were identified among DUEs (Fig. 7), suggesting that DUEs have the potential to alter the activity of the encoding enzymes. Domains functioning in immunity and defense responses were also identified, including protein kinase, lipoxygenase, MLO, and Tify (Fig. 7).

### Differentially used exons impact gall formation and egg mass production

**Figure 8:**
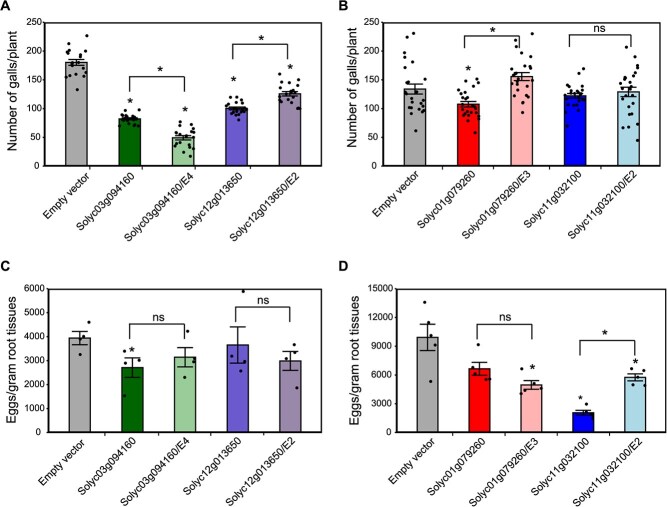
Impact of exon skipping events on plant response to nematode infection. The differentially spliced variants of *Solyc03g094160* (lacking exon 4), *Solyc12g013650* (lacking exon 2), *Solyc01g079260* (lacking exon 3), and *Solyc11g032100* (lacking exon 2) were overexpressed in the susceptible tomato cultivar Heinz 1706 using transgenic hairy root system. Composite plants with GFP positive roots were inoculated with approximately 500 second-stage juveniles of *Meloidogyne incognita.* Six weeks after inoculation, the number of galls per plant (A and B) and number of eggs per gram of root tissues (C and D) were determined. Full-length coding sequences of these four genes were also overexpressed for comparison. Data are presented as mean ± SE. Each dot represents one data point. Statistically significant differences between overexpression and control plants expressing the empty vector were calculated using ANOVA (* P < 0.05).

We examined the functional impact of exon splicing events on plant response to nematode infection by overexpressing splicing variants lacking DUEs using transgenic hairy root system. The overexpression constructs included the coding sequences of *Solyc03g094160* lacking exon 4, *Solyc12g013650* lacking exon 2, *Solyc01g079260.4.1* lacking exon 3, and *Solyc11g032100.2* lacking exon 2. In all cases, full-length coding sequences of these genes were also overexpressed for comparison. In addition, transgenic hairy roots expressing the empty vector were generated and included as control. Transgenic hairy roots were identified using GFP screens, and the composite plants were then inoculated with *M. incognita* second-stage juveniles. The functionality of the overexpression constructs was confirmed by quantifying mRNA abundance of the overexpressed genes in three biological samples randomly collected from different GFP-positive hairy roots. Approximately, 6 weeks after inoculation number of galls per root system as well as number of eggs per gram root tissues were determined. Overexpression of both full-length and splicing variants of *Solyc03g094160* negatively impacted the number of galls as compared with control plants expressing the empty vector (Fig. 8A). However, the splicing variant produced significantly lower number of galls as compared with the full-length variant (Fig. 8A). Overexpression of the full-length variant resulted in 54.4% reduction in number of galls, whereas the splicing variant showed 73% reduction in comparison with control plants (Fig. 8A). Both variants showed similar impact on egg number (Fig. 8C).

In comparison with control plants expressing the empty vector, both full-length and the splicing variant of *Solyc12g013650* significantly reduced the number of galls but the full-length variant produced significantly less galls compared with the splicing variant (Fig. 8A). Both variants showed similar non-significant reduction in egg numbers relative to control plants expressing the empty vector (Fig. 8C). Transgenic hairy roots overexpressing the full-length and splicing variants of *Solyc01g079260* exhibited distinct impacts on gall and egg numbers when compared with control plants (Fig. 8B and D). While the full-length variant produced significantly less gall, the splicing variant produced gall number similar to that of control plant (Fig. 8B). Only the splicing variant significantly reduced the number of eggs as compared with control plants (Fig. 8D). Both full-length and splicing variants of *Solyc11g032100.2* produced similar number of galls per root system, but the number of eggs determined on the transgenic roots overexpressing the splicing variant were twice the number determined on the transgenic roots overexpressing the full-length variant (Fig. 8B and D). Taken together, these data support a role of DUEs in gall formation and/or egg mass production.

## Discussion

In this study, we investigated local and systemic transcriptome and spliceome reprogramming in the *M. incognita*–induced galls and neighboring root cells, and revealed several key features of this reprogramming. *M. incognita* induced substantial transcriptome changes in tomato roots both locally in galls and systemically in neighboring cells at early and late stages of infection. Previous studies documenting transcriptome changes using whole roots as experimental materials in response to *M. incognita* infection suggest a systemic induction of gene expression changes [5–14]. However, the characteristics and magnitude of this induction were not previously reported. Our finding that 63.5% of the DEGs identified in galls at 4 dpi were similarly regulated in neighboring root tissues indicates that this systemic regulation is of biological significance particularly at the early stage of infection. However, at the later stages of infection, the parallel regulation of gene expression in gall and neighboring root cells seems to be less profound as only 19% of the DEGs identified in galls at 11 dpi were similarly regulated in neighboring cells. Cell-to-cell communication during plant development are generally mediated through various signaling mechanisms involving reactive oxygen species (ROS), small peptides, phytohormone signaling and the movement of transcription factors and small non-coding RNAs [28, 29]. The involvement of these signaling mechanisms in establishing plant-nematode interactions [30–33] supports such a role in effective intercellular communication between galls and neighboring root cells considering a significant number of genes coding for these signaling components are identified as differentially expressed.

Coordinated intercellular communications between plant cells is required for proper plant responses to infection relating to defense responses and resource allocation [34]. In this regard, our analysis provided new insights into the coordinated cell-to-cell communications between the nematode-induced galls and neighboring root cells. We observed downregulation of a substantial number of basal defense response genes both in galls and neighboring root cells at the 4-dpi time point (Fig. 5). However, at 11 dpi, the downregulation of the large majority of these genes was restricted to gall cells, suggesting a transition of defense suppression from local/systemic state into local state. This hypothesized transition is likely the result of a switch in plant communication system prioritizing the production and transport of nutrients to the nematode feeding cells. Our analysis also provided evidence involving jasmonic acid signaling in the coordinated cell-to-cell communications between galls and neighboring root cells. Notably, auxin and cytokinin signaling genes were enriched among the similarly downregulated genes in galls and neighboring cells at 4 dpi, whereas genes involved in jasmonic acid–mediated signaling pathway were enriched among the similarly upregulated genes, providing suggestive evidence for suppression of auxin and cytokinin signaling in these tissues. This suggestion is supported by our finding that several auxin response factors (ARFs) and two-component response regulators (type-B ARRs), which are involved in auxin- and cytokinin-mediated signaling [35, 36], respectively, were downregulated in galls and neighboring cells at 4 dpi. In contrast, genes encoding auxin/indole-3-acetic acid proteins (Aux/IAAs) and cytokinin oxidases, which inhibit auxin and cytokine signaling, respectively [35, 37, 38], were upregulated. Unlike auxin and cytokinin, the activation of numerous genes involved in jasmonic acid biosynthesis and signaling suggests a role of jasmonic acid and its metabolite methyl jasmonate, produced upon nematode infection, in systemic signal transmission and cell-to-cell communication. The function of JA and MeJA in short- and long-distance signal transmission has been well established in various plant species, including tomato [39, 40]. In this regard, the systemic reprograming of cells adjacent to the infection sites at early infection stage seems to be critical for creating favorable environments for successful nematode infection by facilitating nutrient flow to the giant-cells. The enrichment of genes involved in translation, cellular catabolic process, vesicle-mediated transport, protein folding, targeting, and degradation, organelle organization, and cell communication supports this view.

Plant transcription factors play key regulatory functions controlling various developmental and physiological processes that determine cell identity [41]. Here, we cataloged about 800 differentially expressed transcription factors belonging to various families, expanding the limited list of regulatory factors functioning during *M. incognita –* tomato interactions. Member of the bHLH, bZIP, GATA, ERF, MYB, WRKY, and ZF families constituted a significant portion of these factors. While members of these families have been shown to regulate plant-nematode interactions [42–48]. Combinatorial interactions between TFs belonging to the same or different families may determine the specificity of the regulatory networks required for gall formation and development. Consistent with this notion, various members of the bHLH, MYB, and bZIP families have been found to form homo- and hetero-dimers with proteins from the same or other families that impact their regulatory function and specificity [42, 49–51]. Our analysis also suggests that members of the transcription factor families E2F, GTE, and HD may contribute to gene expression specificity because they exhibited unique expression patterns in galls or neighboring root tissues. Our analysis also revealed that all differentially expressed transcription factors belonging to the TGA family were downregulated. This may be related to the function of these factors in regulating defense-related genes through physical association with NPR1 (NON-EXPRESSOR OF PR1), a key regulator of immunity [52]. These factors also function in plant immunity in an NPR1-independent manner [53, 54]. Notably, several Trihelix- and Scarecrow-like transcription factors exhibited distinct and overlapping expression patterns in galls and neighboring cells, suggesting that spatial and temporal activities of these factors may contribute to nematode infection success. The potential functional importance of these factors is supported by the findings that members of these families were targeted by *M. incognita* effectors [55, 56].

Unlike the TGA family members, we found that all differentially expressed transcription factors of the E2F family were upregulated, indicative of cell cycle activation during nematode parasitism, considering the fundamental functions of these factors in regulating genes involved in cell cycle progression and DNA synthesis [57]. This indication was further supported by the enrichment of genes involved in mitotic cell cycle among the 11-dpi gall specific DEGs in which 66 out of 72 genes were upregulated. This is consistent with previous findings showing activation of several core cell cycle genes in nematode induced-feeding sites and galls [58–64]. The functional distribution of these genes across various cell cycle stages and checkpoints (Fig. 4) shows how *M. incognita* exerts tight control over the cell cycle process as a whole during infection that results in an increase of ploidy levels in giant-cells and accelerated mitotic activity of surrounding cells. Interestingly, among the cell cycle-related genes we identified several involved in cell cycle checkpoints including, for example, cyclin-dependent kinases, AURORA kinases, and the mitotic checkpoint proteins BUBR1, BUB1 and BUB3. The abundance and differential regulation of these genes may play roles in switching ordinary mitotic cell cycle in giant-cells to aberrant cell cycle involving acytokinetic mitosis and endoreduplication to increase nuclear ploidy levels required for giant-cell ontogeny and enlargement [65, 66].

Our analysis of AS events in galls and adjacent root tissues revealed key features of spliceome reprograming during *M. incognita* infection. First, spliceome reprograming induced by *M. incognita* is profound accounting for 9064 events from 2898 genes. Second, *M. incognita* modulates pre-mRNA splicing at 4 dpi to a much higher degree compared to the 11-dpi time point (8852 events vs 2536). This is consistent with the concept that AS is dynamic in actively dividing cells and differentiating tissues [18, 67, 68]. However, we found that AS events in adjacent root tissues were more prevalent than in galls (8183 events vs 3205). This indicates that cells adjacent to *M. incognita*-induced galls undergo massive post-transcriptional programming involving notable AS, highlighting the essential roles of these cells in establishing the compatibility of the interactions. Third, we found that 52% (1179 events) of the AS events detected in galls at 4 dpi were also identified in the adjacent root cells but this overlap was diminished to only 16% at the 11-dpi time point. This finding indicates that, similar the transcriptome programming, AS in galls and adjacent root tissues are highly coordinated specifically at the early stages of infection despite the mechanism underlying this coordination remain to be determined. Fourth, *M. incognita*–induced AS appears to be linked to gene expression levels particularly in galls. For example, 58.7% and 54% of the DSGs identified in galls at 4 and 11 dpi, respectively, were differentially expressed. In adjacent root cell, this percentage dropped to 48.4% and 14.5% for the 4 and 11 dpi, respectively. A similar association has been previously reported [18]. The substantial overlaps between DSGs and DEGs particularly in galls indicate that transcriptome and spliceome reprograming driven by *M. incognita* infection is mechanistically linked. This can be explained by the functional association between the RNA polymerase II (pol II) and splicing factors where pol II elongation rates regulate AS, and splicing factors can reciprocally alter pol II elongation [69]. This association can also be mediated through a set of co-regulated transcription factors and splicing factors or through regulatory factors with dual functions in gene transcription and splicing.

The splicing patterns revealed suppression of IR and exon usage events in galls and to a much higher degree in the neighboring cells at 4 dpi. However, at 11 dpi, this pattern was reversed in the neighboring cells with IR and exon usage events being more abundant as compared with the corresponding non-infected root tissues. These data suggest that suppression of IRs facilitates *M. incognita* infection of tomato. This can be explained by the fact that IR frequently produces premature termination codons [22], and suppression of these events in galls and neighboring cells would reduce the accumulation of non-functional transcripts, thereby enhancing the translation efficiency of functional transcripts, and hence plant susceptibility. In accord with this view, it has been shown that IR events accumulate to a much higher magnitude in the resistant interactions compared with susceptible interactions [70, 71]. Our analysis also suggests that DUEs, which lead to exon skipping events, may contribute to the establishment of plant–nematode interactions. Protein domain analysis revealed that differential usage of exons can alter protein domain arrangement, and subsequently the activity of various enzymes, including protein kinase, lipoxygenase, and peroxidase, for instance. The enrichment of the peroxidase domain among the DUEs is remarkable and suggests that AS of peroxidase-encoding genes plays a central role in the regulation of plant cellular responses to oxidative stress during infection. The importance of exon usage for the establishment of plant–nematode interactions was further validated by dur data showing that overexpression of 4 splicing variants lacking DUEs negatively impacted gall formation and/or egg mass production. These splicing variants may produce protein isoforms with distinct structures, biological functions, cellular localizations, and protein interaction networks that interfere with successful nematode parasitism.

In conclusion, our study has revealed extensive transcriptome and spliceome reprogramming that occurs during the compatible interaction between *M. incognita* and tomato plants, and has pointed to possible mechanisms of action that facilitate this interface. Our analyses also indicate that widespread modulations of gene expression and AS events occurring systemically in cells adjacent to the *M. incognita*-induced galls represent important features of this interaction. Together, our data documented in the current study provide the foundation for future studies to investigate the importance of local and systemic changes in gene expression and AS with respect to gall ontogeny and nematode parasitism.

## Materials and methods

### Plant material and growth conditions

Tomato (*Solanum lycopersicum*) cultivar Heinz 1706 was used in all experiments described in this study. Plants were grown in controlled growth chambers at 26°C under 16-h light/8-h dark conditions.

### Nematode inoculation, tissue collection, and RNA-seq library construction

Seeds were surface-sterilized and germinated in sterile topsoil (Promix BK25 Peat/Bark Based). Then, 2-week-old tomato seedlings were transferred to pots containing a sterilized mixture of sand and topsoil at a 3:1 ratio. Each plant was inoculated with about 300 second-stage juveniles of *M. incognita* or a mock 0.1% agarose solution (control). Inoculated and control plants were organized in a randomized complete block design in a growth chamber at 26°C under 16-h light/8-h dark conditions and 65% humidity. Galls, neighboring regions, and non-infected whole roots were collected at 4- and 11-day post inoculation (dpi) in three biological replicates, resulting in a total of 18 samples. Approximately, 250 galls and neighboring regions were collected for each replicate. Isolation of DNA-free RNA was performed using Direct-zol™ RNA Miniprep Plus Kit (Zymo Research) following the manufacturer's protocol. rRNA-depleted RNA-seq libraries were prepared using rRNA-depleted RNA and a NEBNext Ultra Directional RNA Library Prep Kit for Illumina (New England Biolabs, Beverly, MA, USA) according to the manufacturer’s protocol. The libraries were sequenced using the NovaSeq 6000 platform (Illumina Corp., San Diego, CA, USA) with 150-bp paired-end reads.

### RNA-seq data analysis

Quality assessment of sequenced reads was performed using FastQC software (http://www.bioinformatics.babraham.ac.uk/projects/fastqc/). After removing the low-quality reads and adapter sequences with TRIMMOMATIC, version 0.39.1 [72], the remaining high-quality paired-end reads were mapped to the *S. lycopersicum* genome version SL4.0 using STAR (version 2.7.9a) [73]. Duplicated reads were removed using PICARD (http://broadinstitute.github.io/picard/), version 2.25.2. Reads mapped to the tomato genome were counted using HTSEQ [74], version 0.13.5. Differentially expressed genes (DEGs) were identified using DESEQ2 package [75] version 1.30.1 in R studio using an adjusted P value cutoff of 0.05. Gene annotation we conducted using Heinz 1706 tomato gene descriptions (ITAG4.0), available at https://solgenomics.net/.

### Alternative splicing analysis

Differentially used exons (DUE) and intron retention (IR) events were determined using the R package JUNCTIONSEQ [27], which utilizes QORTS quality-control/data-processing software [76] for read quality control counts. Reads mapped to each non-overlapping exons and splice junctions were counted and used to determine DUE and IR events using DESEQ2 [75] with a false discovery rate (FDR) cut off of 5% as recently described [18].

### Gene ontology enrichment analysis

Gene Ontology (GO) term enrichment analysis of DEGs and DSGs was performed using PANTHER version 18.0 with Fisher’s exact test and Bonferroni multi-test correction with a cut off P-value of 0.01 for significance.

### Generation of transgenic tomato hairy roots and nematode infection assays

The full-length coding sequences as well as differentially spliced variants of *Solyc01g079260* (lacking exon 3), *Solyc11g032100* (lacking exon 2), *Solyc12g013650* (lacking exon 2), and *Solyc03g094160* (lacking exon 4) were synthesized and cloned in the pG2RNAi2 vector under the control of a strong ubiquitin promoter. Primer sequences used for cloning are provided in Supplementary Table S11. The pG2RNAi2 binary vector contains a super folded green fluorescent protein (sGFP) for the identification of transgenic hairy roots. All constructs were confirmed by sequencing, and then were transformed into *Agrobacterium -rhizogenes* strain K599 and used for generating transgenic tomato hairy roots in the susceptible Heinz 1706 background. The roots of 1-week-old seedling were removed and the seedlings were vacuum-infiltrated with agrobacterium suspension (OD600 of 1.0) four times each for 90 seconds. The infiltrated seedlings were maintained under high humidity conditions (65%) in a growth chamber with 16-h light/8-h dark conditions at 26°C for 3 weeks. The generated hairy roots were then screened using dissecting microscope containing GFP filter to identify transgenic hairy roots. Composite plants with GFP-positive roots were planted in pots containing topsoil (Premier Pro-Mix BK25 Peat/Bark Based Growing Soil), and inoculated with approximately 500 freshly hatched second-stage juveniles of *M. incognita.* Six weeks after inoculation, the number of galls per plant and number of eggs per gram of root tissues were determined. The infection experiments were organized in a randomized complete block design with between 15 and 20 replicated plants per construct. Statistically significant differences between overexpression and control plants expressing the empty vectors were calculated using analysis of variance with *P* < 0.05.

### Reverse-transcription quantitative PCR assays

To confirm the functionality of overexpression constructs, total RNA was extracted from transgenic hairy roots overexpressing the full-length coding sequences and differentially spliced variants of *Solyc01g079260*, *Solyc11g032100*, *Solyc12g013650*, and *Solyc03g094160* as previously described [77] and subjected to RT-qPCR quantification using gene specific primers (Supplementary Data Table S11). Total RNA was also extracted from transgenic hairy roots expressing the empty binary vector and used as control. To quantify the expression levels of various splicing events, total RNA was isolated from galls and neighboring root regions at 4 and 11 dpi. Total RNA was also isolated from non-infected root tissues at each both time points and used as control. Primers were designed to specifically amplify the directed intron or exon in each splicing event (Supplementary Data Table S11). RT-qPCR quantifications were conducted using Verso SYBR green 1-step RT-qPCR (Thermo Fisher Scientific), with 20 ng of RNA per reaction, following the manufacturer's instruction. Gene expression values were normalized using *actin* (*Solyc11g005330.2.1*) as a reference gene. Relative fold change values were calculated using three biological samples. Each reaction was performed with three technical replicates. Statistically significant differences between control and overexpression samples were determined by *t* tests (*P* < 0.05).

## Supplementary Material

Web_Material_uhae206

## Data Availability

RNA-sequencing data described in this paper have been submitted to the NCBI database, Gene Expression Omnibus under accession no GSE232828.

## References

[1] Nicol JM, Turner SJ, Coyne DL. et al. Current nematode threats to world agriculture. In: Jones J, Gheysen G, Fenoll C, eds. Genomics and Molecular Genetics of Plant-Nematode Interactions. Springer: New York, NY, 2011,3–20

[2] Hunt DJ, Handoo ZA. Taxonomy, identification and principal species. In: Perry RN, Moens M, Starr JL (eds.), Root-Knot Nematodes. CABI: Wallingford UK, 2009,55–97

[3] Escobar C, Barcala M, Cabrera J. et al. Overview of root-knot nematodes and giant cells. Adv Bot Res. 2015;73:1–32

[4] Hewezi T, Baum TJ. Communication of sedentary plant-parasitic nematodes with their host plants. Adv Bot Res. 2017;82:305–24

[5] Jammes F, Lecomte P, De Almeida-Engler J. et al. Genome-wide expression profiling of the host response to root-knot nematode infection in Arabidopsis. Plant J. 2005;44:447–58 16236154 10.1111/j.1365-313X.2005.02532.x

[6] Fuller VL, Lilley CJ, Atkinson HJ. et al. Differential gene expression in Arabidopsis following infection by plant-parasitic nematodes Meloidogyne incognita and *Heterodera schachtii*. Mol Plant Pathol. 2007;8:595–609 20507524 10.1111/j.1364-3703.2007.00416.x

[7] Schaff JE, Nielsen DM, Smith CP. et al. Comprehensive transcriptome profiling in tomato reveals a role for glycosyltransferase in Mi-mediated nematode resistance. Plant Physiol. 2007;144:1079–92 17434994 10.1104/pp.106.090241 PMC1914198

[8] Postnikova OA, Hult M, Shao J. et al. Transcriptome analysis of resistant and susceptible alfalfa cultivars infected with root-knot nematode *Meloidogyne incognita*. PLoS One. 2015;10:e0118269 25710378 10.1371/journal.pone.0118269 PMC4339843

[9] Santini L, De Freitas Munhoz C, Bonfim MF. et al. Host transcriptional profiling at early and later stages of the compatible interaction between *Phaseolus vulgaris* and *Meloidogyne incognita*. Phytopathology. 2016;106:282–94 26551451 10.1094/PHYTO-07-15-0160-R

[10] Shukla N, Yadav R, Kaur P. et al. Transcriptome analysis of root-knot nematode (*Meloidogyne incognita*)-infected tomato (*Solanum lycopersicum*) roots reveals complex gene expression profiles and metabolic networks of both host and nematode during susceptible and resistance responses. Mol Plant Pathol. 2018;19:615–33 28220591 10.1111/mpp.12547 PMC6638136

[11] Li X, Xing X, Tian P. et al. Comparative transcriptome profiling reveals defense-related genes against *Meloidogyne incognita* invasion in tobacco. Molecules. 2018;23:2081 30127271 10.3390/molecules23082081 PMC6222693

[12] Lee IH, Kim HS, Nam KJ. et al. The defense response involved in sweet potato resistance to root-knot nematode *Meloidogyne incognita*: comparison of root transcriptomes of resistant and susceptible sweet potato cultivars with respect to induced and constitutive defense responses. Front Plant Sci. 2021;12:671677 34025707 10.3389/fpls.2021.671677 PMC8131533

[13] Li X, Sun Y, Yang Y. et al. Transcriptomic and histological analysis of the response of susceptible and resistant cucumber to *Meloidogyne incognita* infection revealing complex resistance via multiple signaling pathways. Front Plant Sci. 2021;12:675429 34194451 10.3389/fpls.2021.675429 PMC8236822

[14] Sung YW, Kim J, Yang JW. et al. Transcriptome-based comparative expression profiling of sweet potato during a compatible response with root-knot nematode *Meloidogyne incognita* infection. Genes (Basel). 2023;14:2074 38003017 10.3390/genes14112074 PMC10671793

[15] Filichkin S, Priest HD, Megraw M. et al. Alternative splicing in plants: directing traffic at the crossroads of adaptation and environmental stress. Curr Opin Plant Biol. 2015;24:125–35 25835141 10.1016/j.pbi.2015.02.008

[16] Laloum T, Martín G, Duque P. Alternative splicing control of abiotic stress responses. Trends Plant Sci. 2018;23:140–50 29074233 10.1016/j.tplants.2017.09.019

[17] Rigo R, Bazin J, Crespi M. et al. Alternative splicing in the regulation of plant–microbe interactions. Plant Cell Physiol. 2019;60:1906–16 31106828 10.1093/pcp/pcz086

[18] Niyikiza D, Piya S, Routray P. et al. Interactions of gene expression, alternative splicing, and DNA methylation in determining nodule identity. Plant J. 2020;103:1744–66 32491251 10.1111/tpj.14861

[19] Kufel J, Diachenko N, Golisz A. Alternative splicing as a key player in the fine-tuning of the immunity response in Arabidopsis. Mol Plant Pathol. 2022;23:1226–38 35567423 10.1111/mpp.13228 PMC9276941

[20] Hewezi T . Phytopathogens reprogram host alternative mRNA splicing. Annu Rev Phytopathol. 2024;62: ahead of print 10.1146/annurev-phyto-121423-041908 38691872

[21] Sun B, Huang J, Gao C. et al. Alternative splicing of a potato disease resistance gene maintains homeostasis between development and immunity, and functions as a novel process for pathogen surveillance. Plant Cell. 2024;koae189 10.1093/plcell/koae189 PMC11371151 38941447

[22] Reddy ASN, Marquez Y, Kalyna M. et al. Complexity of the alternative splicing landscape in plants. Plant Cell. 2013;25:3657–83 24179125 10.1105/tpc.113.117523 PMC3877793

[23] Verma A, Lee C, Morriss S. et al. The novel cyst nematode effector protein 30D08 targets host nuclear functions to alter gene expression in feeding sites. New Phytol. 2018;219:697–713 29726613 10.1111/nph.15179

[24] Mejias J, Bazin J, Truong N-M. et al. The root-knot nematode effector MiEFF18 interacts with the plant core spliceosomal protein SmD1 required for giant cell formation. New Phytol. 2021;229:3408–23 33206370 10.1111/nph.17089

[25] Rambani A, Pantalone V, Yang S. et al. Identification of introduced and stably inherited DNA methylation variants in soybean associated with soybean cyst nematode parasitism. New Phytol. 2020;227:168–84 32112408 10.1111/nph.16511

[26] Hawk TE, Piya S, Zadegan SB. et al. The soybean immune receptor GmBIR1 regulates host transcriptome, spliceome, and immunity during cyst nematode infection. New Phytol. 2023;239:2335–52 37337845 10.1111/nph.19087

[27] Hartley SW, Mullikin JC. Detection and visualization of differential splicing in RNA-Seq data with JunctionSeq. Nucleic Acids Res. 2016;44:e127 27257077 10.1093/nar/gkw501 PMC5009739

[28] van Norman JM, Breakfield NW, Benfey PN. Intercellular communication during plant development. Plant Cell. 2011;23:855–64 21386031 10.1105/tpc.111.082982 PMC3082268

[29] Jin J, Yu S, Lu P. et al. Deciphering plant cell–cell communications using single-cell omics data. Comput Struct Biotechnol J. 2023;21:3690–5 37576747 10.1016/j.csbj.2023.06.016 PMC10412842

[30] Gheysen G, Mitchum MG. Phytoparasitic nematode control of plant hormone pathways. Plant Physiol. 2019;179:1212–26 30397024 10.1104/pp.18.01067 PMC6446774

[31] Sato K, Kadota Y, Shirasu K. Plant immune responses to parasitic nematodes. Front Plant Sci. 2019;10:1165 31616453 10.3389/fpls.2019.01165 PMC6775239

[32] Hewezi T . Epigenetic mechanisms in nematode–plant interactions. Annu Rev Phytopathol. 2020;58:119–38 32413274 10.1146/annurev-phyto-010820-012805

[33] Mitchum MG, Liu X. Peptide effectors in phytonematode parasitism and beyond. Annu Rev Phytopathol. 2022;60:97–119 35385672 10.1146/annurev-phyto-021621-115932

[34] Biere A, Goverse A. Plant-mediated systemic interactions between pathogens, parasitic nematodes, and herbivores above- and belowground. Annu Rev Phytopathol. 2016;54:499–527 27359367 10.1146/annurev-phyto-080615-100245

[35] Chandler JW . Auxin response factors. Plant Cell Environ. 2016;39:1014–28 26487015 10.1111/pce.12662

[36] Kieber JJ, Schaller GE. Cytokinin signaling in plant development. Development. 2018;145:dev149344 29487105 10.1242/dev.149344

[37] Werner T, Köllmer I, Bartrina I. et al. New insights into the biology of cytokinin degradation. Plant Biol. 2006;8:371–81 16807830 10.1055/s-2006-923928

[38] Piya S, Shrestha SK, Binder B. et al. Protein-protein interaction and gene co-expression maps of ARFs and Aux/IAAs in Arabidopsis. Front Plant Sci. 2014;5:744 25566309 10.3389/fpls.2014.00744 PMC4274898

[39] Heil M, Ton J. Long-distance signalling in plant defence. Trends Plant Sci. 2008;13:264–72 18487073 10.1016/j.tplants.2008.03.005

[40] Ruan J, Zhou Y, Zhou M. et al. Jasmonic acid signaling pathway in plants. Int J Mol Sci. 2019;20:2479 31137463 10.3390/ijms20102479 PMC6566436

[41] Xu X, Smaczniak C, Muino JM. et al. Cell identity specification in plants: lessons from flower development. J Exp Bot. 2021;72:4202–17 33865238 10.1093/jxb/erab110 PMC8163053

[42] Jin J, Hewezi T, Baum TJ. The Arabidopsis bHLH25 and bHLH27 transcription factors contribute to susceptibility to the cyst nematode *Heterodera schachtii*. Plant J. 2011;65:319–28 21223395 10.1111/j.1365-313X.2010.04424.x

[43] Piya S, Kihm C, Hollis Rice J. et al. Cooperative regulatory functions of miR858 and MYB83 during cyst nematode parasitism. Plant Physiol. 2017;174:1897–912 28512179 10.1104/pp.17.00273 PMC5490899

[44] Xu X, Fang P, Zhang H. et al. Strigolactones positively regulate defense against root-knot nematodes in tomato. J Exp Bot. 2019;70:1325–37 30576511 10.1093/jxb/ery439 PMC6382333

[45] Kumar A, Sichov N, Bucki P. et al. SlWRKY16 and SlWRKY31 of tomato, negative regulators of plant defense, involved in susceptibility activation following root-knot nematode *Meloidogyne javanica* infection. Sci Rep. 2023;13:14592 37669955 10.1038/s41598-023-40557-z PMC10480479

[46] Huang H, Zhao W, Qiao H. et al. SlWRKY45 interacts with jasmonate-ZIM domain proteins to negatively regulate defense against the root-knot nematode Meloidogyne incognita in tomato. Hortic Res. 2022;9:uhac197 36338841 10.1093/hr/uhac197 PMC9630973

[47] Ribeiro C, de Melo BP, Lourenço-Tessutti IT. et al. The regeneration conferring transcription factor complex ERF115-PAT1 coordinates a wound-induced response in root-knot nematode induced galls. New Phytol. 2024;241:878–95 38044565 10.1111/nph.19399

[48] Liu X, Mitchum MG. Title: a major role of class III HD-ZIPs in promoting sugar beet cyst nematode syncytium. bioRxiv. 2024 10.1371/journal.ppat.1012610 PMC11542791 39509386

[49] Dubos C, Stracke R, Grotewold E. et al. MYB transcription factors in Arabidopsis. Trends Plant Sci. 2010;15:573–81 20674465 10.1016/j.tplants.2010.06.005

[50] Alves MS, Dadalto SP, Gonçalves AB. et al. Plant bZIP transcription factors responsive to pathogens: a review. Int J Mol Sci. 2013;14:7815–28 23574941 10.3390/ijms14047815 PMC3645718

[51] Pireyre M, Burow M. Regulation of MYB and bHLH transcription factors: a glance at the protein level. Mol Plant. 2015;8:378–88 25667003 10.1016/j.molp.2014.11.022

[52] Zhang Y, Fan W, Kinkema M. et al. Interaction of NPR1 with basic leucine zipper protein transcription factors that bind sequences required for salicylic acid induction of the PR-1 gene. Proc Natl Acad Sci. 1999;96:6523–8 10339621 10.1073/pnas.96.11.6523 PMC26915

[53] Gatz C . From pioneers to team players: TGA transcription factors provide a molecular link between different stress pathways. Mol Plant-Microbe Interact. 2013;26:151–9 23013435 10.1094/MPMI-04-12-0078-IA

[54] Tomaž Š, Gruden K, Coll A. TGA transcription factors—structural characteristics as basis for functional variability. Front Plant Sci. 2022;13:935819 35958211 10.3389/fpls.2022.935819 PMC9360754

[55] Huang G, Dong R, Allen R. et al. A root-knot nematode secretory peptide functions as a ligand for a plant transcription factor. Mol Plant-Microbe Interact. 2006;19:463–70 16673933 10.1094/MPMI-19-0463

[56] Zhao J, Huang K, Liu R. et al. The root-knot nematode effector Mi2G02 hijacks a host plant trihelix transcription factor to promote nematode parasitism. Plant Commun. 2024;5:100723 37742073 10.1016/j.xplc.2023.100723 PMC10873892

[57] Del Pozo JC, Diaz-Trivino S, Cisneros N. et al. The balance between cell division and endoreplication depends on E2FC-DPB, transcription factors regulated by the ubiquitin-SCFSKP2A pathway in Arabidopsis. Plant Cell. 2006;18:2224–35 16920782 10.1105/tpc.105.039651 PMC1560920

[58] Niebel A, De Almeida Engler J, Hemerly A. et al. Induction of cdc2a and cyc1At expression in Arabidopsis thaliana during early phases of nematode-induced feeding cell formation. Plant J. 1996;10:1037–43 9011085 10.1046/j.1365-313x.1996.10061037.x

[59] De Almeida Engler J, De Vleesschauwer V, Burssens S. et al. Molecular markers and cell cycle inhibitors show the importance of cell cycle progression in nematode-induced galls and syncytia. Plant Cell. 1999;11:793–807 10330466 10.1105/tpc.11.5.793 PMC144216

[60] Favery B, Complainville A, Vinardell JM. et al. The endosymbiosis-induced genes ENOD40 and CCS52a are involved in endoparasitic–nematode interactions in *Medicago Truncatula*. Mol Plant-Microbe Interact. 2002;15:1008–13 12437298 10.1094/MPMI.2002.15.10.1008

[61] De Almeida Engler J, Kyndt T, Vieira P. et al. CCS52 and DEL1 genes are key components of the endocycle in nematode-induced feeding sites. Plant J. 2012;72:185–98 22640471 10.1111/j.1365-313X.2012.05054.x

[62] De Almeida Engler J, Gheysen G. Nematode-induced endoreduplication in plant host cells: why and how? Mol Plant-Microbe Interact. 2013;26:17–24 23194340 10.1094/MPMI-05-12-0128-CR

[63] Vieira P, De Clercq A, Stals H. et al. The cyclin-dependent kinase inhibitor KRP6 induces mitosis and impairs cytokinesis in giant cells induced by plant-parasitic nematodes in Arabidopsis. Plant Cell. 2014;26:2633–47 24963053 10.1105/tpc.114.126425 PMC4114956

[64] Vieira P, Escudero C, Rodiuc N. et al. Ectopic expression of Kip-related proteins restrains root-knot nematode-feeding site expansion. New Phytol. 2013;199:505–19 23574394 10.1111/nph.12255

[65] Caillaud MC, Paganelli L, Lecomte P. et al. Spindle assembly checkpoint protein dynamics reveal conserved and unsuspected roles in plant cell division. PLoS One. 2009;4:e6757 19710914 10.1371/journal.pone.0006757 PMC2728542

[66] de Almeida Engler J, Vieira P, Rodiuc N. et al. The plant cell cycle machinery: usurped and modulated by plant-parasitic nematodes. Adv Bot Res. 2015;73:91–118

[67] Shen Y, Zhou Z, Wang Z. et al. Global dissection of alternative splicing in paleopolyploid soybean. Plant Cell. 2014;26:996–1008 24681622 10.1105/tpc.114.122739 PMC4001406

[68] Thatcher SR, Zhou W, Leonard A. et al. Genome-wide analysis of alternative splicing in Zea mays: landscape and genetic regulation. Plant Cell. 2014;26:3472–87 25248552 10.1105/tpc.114.130773 PMC4213170

[69] Kornblihtt AR, De La Mata M, Fededa JP. et al. Multiple links between transcription and splicing. RNA. 2004;10:1489–98 15383674 10.1261/rna.7100104 PMC1370635

[70] Howard BE, Hu Q, Babaoglu AC. et al. High-throughput RNA sequencing of pseudomonas-infected Arabidopsis reveals hidden transcriptome complexity and novel splice variants. PLoS One. 2013;8:e74183 24098335 10.1371/journal.pone.0074183 PMC3788074

[71] Laskar P, Hazra A, Pal A. et al. Deciphering the role of alternative splicing as modulators of defense response in the MYMIV–Vigna mungo pathosystem. Physiol Plant. 2023;175:e13922 37114622 10.1111/ppl.13922

[72] Bolger AM, Lohse M, Usadel B. Trimmomatic: a flexible trimmer for Illumina sequence data. Bioinformatics. 2014;30:2114–20 24695404 10.1093/bioinformatics/btu170 PMC4103590

[73] Dobin A, Davis CA, Schlesinger F. et al. STAR: ultrafast universal RNA-seq aligner. Bioinformatics. 2013;29:15–21 23104886 10.1093/bioinformatics/bts635 PMC3530905

[74] Anders S, Pyl PT, Huber W. HTSeq-A python framework to work with high-throughput sequencing data. Bioinformatics. 2015;31:166–9 25260700 10.1093/bioinformatics/btu638 PMC4287950

[75] Love MI, Huber W, Anders S. Moderated estimation of fold change and dispersion for RNA-seq data with DESeq2. Genome Biol. 2014;15:550 25516281 10.1186/s13059-014-0550-8 PMC4302049

[76] Hartley SW, Mullikin JC. QoRTs: a comprehensive toolset for quality control and data processing of RNA-Seq experiments. BMC Bioinformatics. 2015;16:224 26187896 10.1186/s12859-015-0670-5 PMC4506620

[77] Verwoerd T, Dekker B, Hoekema A. Nucleic acids research a small-scale procedure for the rapid isolation of plant RNAs. Nucleic Acids Res. 1989;17:2362 2468132 10.1093/nar/17.6.2362 PMC317610

