# Correction to: Local and systemic transcriptome and spliceome reprogramming induced by the root-knot nematode *Meloidogyne incognita* in tomato

**DOI:** 10.1093/hr/uhaf056

**Published:** 2025-03-04

This is a correction to: Selin Ozdemir *et al.*, Local and systemic transcriptome and spliceome reprogramming induced by the root-knot nematode *Meloidogyne incognita* in tomato, *Horticulture Research*, Volume 11, Issue 9, September 2024, uhae206, 10.1093/hr/uhae206.

*M. incognita* was incorrectly expanded to read *Megalaima incognita*. This has been corrected to read *Meloidogyne incognita*. The Publisher apologizes for the error.

