# Supplementary material for: Local and systemic transcriptome and spliceome reprogramming induced by the root-knot nematode Meloidogyne incognita in tomato
Source: Hortic Res. 2024 Jul 26;11(9):uhae206. doi: 10.1093/hr/uhae206 (PMC11403207; doi:10.1093/hr/uhae206)
Supplement: Web_Material_uhae206 [file web_material_uhae206.zip › Supplementary Figure S1.pdf]

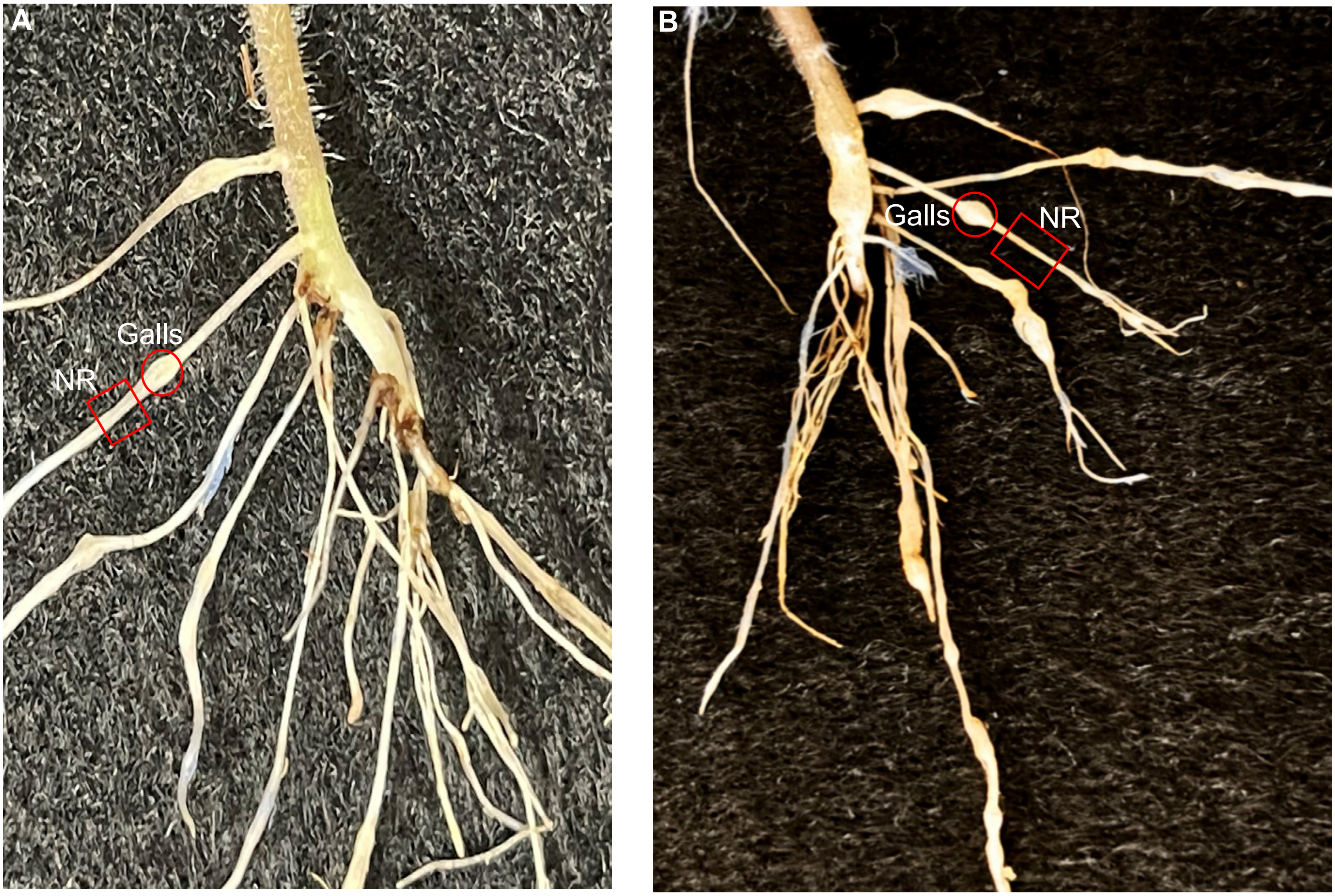

Supplementary Fig. S1: Images showing examples of dissected galls and neighboring regions at 4- and 11-days post *M. incognita* infection.

A and B: Dissected galls and neighboring regions (NR) at 4 (A) and 11 (B) days post *M. incognita* infection.
