# Supplementary material for: Local and systemic transcriptome and spliceome reprogramming induced by the root-knot nematode Meloidogyne incognita in tomato
Source: Hortic Res. 2024 Jul 26;11(9):uhae206. doi: 10.1093/hr/uhae206 (PMC11403207; doi:10.1093/hr/uhae206)
Supplement: Web_Material_uhae206 [file web_material_uhae206.zip › Supplementary Figure S2.pdf]

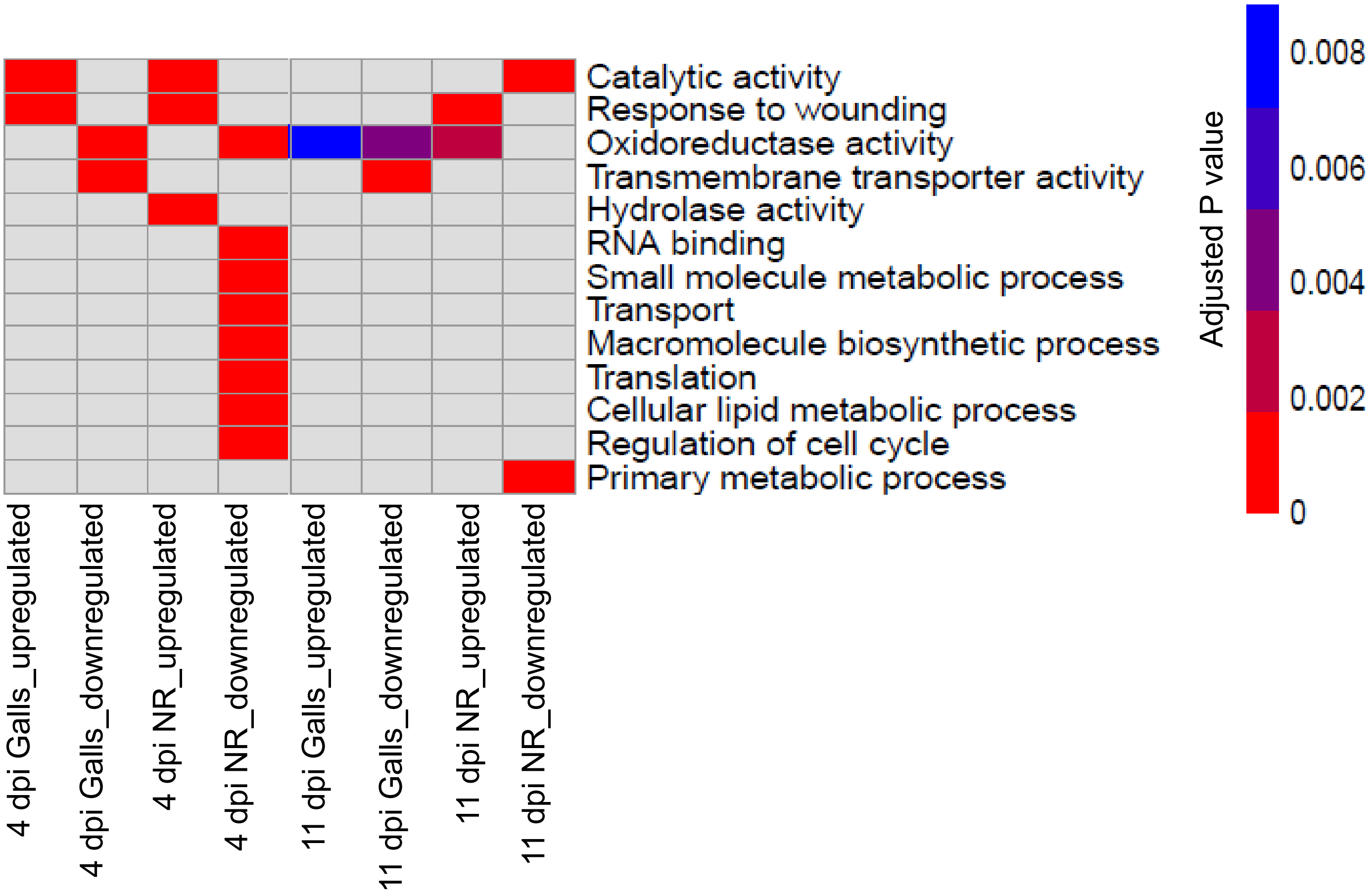

Supplementary Fig. S2: Significantly enriched GO terms among differentially spliced genes identified in galls and neighboring root cells (NR) at 4- and 11-days post *M. incognita* infection.

Gene Ontology (GO) term enrichment analysis of differentially spliced genes (DSGs) was performed using PANTHER with Fisher's exact test and Bonferroni multi-test for correction with a cut off P-value of 0.01 for significance.
